# Pectin Edible Films Filled with *Ilex paraguariensis* Concentrate Extract and Its Characterization

**DOI:** 10.3390/polym16223158

**Published:** 2024-11-13

**Authors:** Carolina Aparecida Antunes Amadeu, Francielli Brondani Silva, Clitor Júnior Fernandes Souza, Marivane Turim Koschevic, Vanderleia Schoeninger, Evaristo Alexandre Falcão, Vitor Augusto Dos Santos Garcia, Claudia Andrea Lima Cardoso, Silvia Maria Martelli

**Affiliations:** 1 Faculty of Engineering, Federal University of Grande Dourados, Dourados 79825-070, MS, Brazil; caruh.antunes@hotmail.com (C.A.A.A.); franbrondani@hotmail.com (F.B.S.); clitorrj@gmail.com (C.J.F.S.); 2 Faculty of Exact Sciences and Technology, Federal University of Grande Dourados, Dourados 79825-070, MS, Brazil; marivane.koschevic@gmail.com (M.T.K.); evaristofalcao@ufgd.edu.br (E.A.F.); 3 Faculty of Agrarian Sciences, Federal University of Grande Dourados, Dourados 79825-070, MS, Brazil; vschoeninger@ufgd.edu.br; 4 Faculty of Agricultural Sciences, São Paulo State University (UNESP), Avenue Universitária, Botucatu 18610-034, SP, Brazil; vitor.as.garcia@unesp.br; 5 Center Studies in Natural Resources, State University of Mato Grosso do Sul, Dourados 79804-970, MS, Brazil; claudiacardosouems1@gmail.com

**Keywords:** functional film, experimental design, yerba mate, antioxidant compounds, green technology

## Abstract

*Ilex paraguariensis* (IP) extract was added to prepare edible films using a central rotational composite design (CCRD) 2^2^ with IP extract and sorbitol concentrations as variables. The IP extract was characterized by color parameters, total phenolic content, caffeine, flavonoids, and chlorophyll content, and antioxidant activity and the edible films were assessed for the same analysis and thickness, water vapor permeability (WVP), solubility in water, fluorescence, photodegradation and UV/Vis light barrier, FT-IR, thermogravimetry, and differential exploratory colorimetry. Sorbitol increased thickness and WVP, while the extract influenced the concentration of phenolic compounds in the films. The optimum concentrations of extract and sorbitol were 10% and 15%, respectively. Films presented thermal resistance (until 230 °C) and an excellent barrier to UV light. Furthermore, these films could carry compounds originally in IP, showing good functional properties concerning the water vapor barrier (showing a great variation scale due to the possibility to increase sorbitol or not, between 3.33 and 5.27 g mm/m^2^ day KPa). The films showed great potential to replace conventional primary packaging, and if consumed with food, as a bullet paper, they can add nutritional value to the packaged product.

## 1. Introduction

Interest in a balanced life along with the scientific evidence that functional foods have health benefits have made consumers choose wholesome marketed products that provide healthy characteristics [1,2]. There are significant changes in the consumer’s profile, where the increase in consumption of health services and products shows the growing search for an improved quality of life [3], with products in the food segment being the most evident. Moreover, the growing concern with environmental preservation has promoted the search for environmentally less aggressive technologies.

Among the new technologies, the use of biodegradable films and coatings are excellent examples because they are non-toxic and safe, non-polluting, simple to apply, and low cost to obtain the raw materials and processing, while also providing beneficial effects such as good sensory attributes, high barrier power, good mechanical properties, and microbial stability [4,5,6]. Their properties positively affect the packaged product and the environment, as they are generally compatible and biodegradable [7].

The main components of edible films are polysaccharides, proteins, and lipids. Among polysaccharides are starch, chitosan, alginate, pectins, and cellulose derivatives. Pectin is an edible polymer recognized as safe for human consumption (GRAS—generally recognized as safe) by the FDA (Food and Drug Administration—USA) [8]. This polymer is biocompatible, non-toxic, degraded by bacteria, highly available in nature, low cost, highly stable, has suitable gelling properties, and provides easy chemical and biochemical modification [9,10,11,12].

For the production of edible films, in addition to polymers (filmogenic agents), the use of plasticizers in the production process is necessary. Sorbitol and glycerol are the most employed plasticizers because they improve flexibility and handling, maintain the integrity, and reduce the number of pores and cracks in the polymeric matrix that can be formed during the coating process [13].

Recent research and publications about edible films and coatings production has focused on different substances or compounds such as antioxidants, antimicrobials, colorants or flavorings, and nutraceuticals [14,15,16,17,18,19]. These compounds were incorporated into the film matrix to enhance physical properties, give a new function as active and intelligent packaging, and make the packaged food functional. Through the incorporation in edible films of active compounds obtained from natural extracts, mainly antioxidants and antimicrobials, edible films can influence the quality of foods and extend their shelf life. 

Pectin edible films have been enriched with *Morus alba* leaf extract and showed that incorporation of crude mulberry leaf extract and bioactive components derived from them has greater biocompatibility and significantly improved mechanical and water barrier properties, which supports their suitability for food packaging/coating applications [20]. Pectin edible films have also been incorporated with several antimicrobial substances such as nisin and essential oils to obtain antimicrobial active packaging that contributes to extending product shelf life and reducing the risk of pathogen growth on food surfaces [9,21,22]. Several plants are used for the obtention of extracts and application in edible films; among them, we can highlight the *Ilex paraguariensis*, popularly known as Erva Mate or Mate in Brazil and Yerba Mate or Paraguayan tea in Paraguay. It is a species from the family Aquifoliaceae and genus *Ilex*, which comprises approximately 600 species [23]. The chemical composition of this plant is variable; it contains xanthines, saponins, phenolic compounds, catechins, amino acids, flavonoids, minerals, caffeine, and vitamins C, B1, and B2, thus being an excellent additive [24,25,26].

Therefore, this study aims to develop edible and biodegradable films based on pectin and sorbitol added with a characterized dried extract of *Ilex paraguariensis*, for later application as primary food packaging.

## 2. Materials and Methods

### 2.1. Materials

The extract was obtained from the *Ilex paraguariensis* (IP) “extra strong” type, purchased in Dourados (MS, Brazil). The citrus pectin and D-sorbitol P.S. (PM: 182.17) were both from Dinamic^®^ (São Paulo, Brazil). Anhydrous ethyl acetate (99.8%), methanol, and ethanol standards (99.5%) (Merck, São Paulo, Brazil) were of analytical grade. All solutions used for the extractions were prepared using ultra-pure water.

### 2.2. Ilex paraguariensis (IP) Extract

The IP powder was separated by a 600 µm vibrate sieve in order to separate the stems and large leaves, to increase the contact surface with the solvent, and improve the concentration. The extract was prepared with 3% (*w*/*v* 3 g/100 mL) of IP in 25% (*v*/*v*) methanol in water, and the solvent and its concentration was determined from the analyses performed by Ribani (2006) [27]. The sample was mechanically shaken in a Shaker (TECNAL^®^, TE-420, São Paulo, Brazil) at 200 rpm for 20 min and then sonicated (Unique manual) for 20 min to optimize extraction. Both processes were carried out at room temperature. Subsequently, the solution was filtered on nonwoven fabric (weight: 40 g/m^2^) and centrifuged for 3 min. The supernatant was destined for the rotary evaporator (Fisatom-802, São Paulo, Brazil) until complete evaporation of methanol, and the resulting solution was distributed on glass plates and submitted to air-circulation (PRISMA Lab) at 35 ± 2 °C for approximately 20 h for the evaporation of residual water.

### 2.3. Characterization of Ilex paraguariensis Extract 

#### 2.3.1. Color Parameters 

The color analysis (L*, chroma a*, and chroma b*) was performed by reading the color directly on the surface of *Ilex paraguariensis* leaves powder and on the *Ilex paraguariensis* dried extract placed in a glass petri dish, using the digital colorimeter CR 400 (Konica Minolta, São Paulo, Brazil).

#### 2.3.2. Total Phenolic Content and Caffeine Content

The content of total phenolic compounds was determined by the Folin–Ciocalteu reagent [28]. Gallic acid was the reference standard, and distilled water was the solvent for all samples. The results were obtained after calculating and correcting the extract or herb mass and solvent volume. 

The caffeine content was quantified by the spectrophotometric method. Caffeine stock solution (100 ppm) was prepared by dissolving 0.01 g of recrystallized caffeine in 100 mL of chloroform. The following dilutions were prepared from the caffeine stock solution: 1, 5, 10, 15, 20, and 25 ppm. Their absorbances were measured at 274 nm in quartz cuvettes three times for each dilution. The absorbance values were used for the calibration of caffeine content analysis. Exactly 2 g of IP extract was weighed, and 20 mL of distilled water was added to the sample. The content was heated and then boiled for 10 min. A total of 2 g of sodium carbonate was added to each sample for tannin precipitation. Samples were filtered, and filtrates were concentrated at 5 mL by heating. From the given volume, caffeine was extracted by adding 5 mL of chloroform in the separatory funnel. Caffeine was extracted by stirring in the separatory funnel for a few minutes. The lower caffeine-containing layer was separated and analyzed for caffeine content with UV/Vis spectrophotometer. An amount of 0.1 mL of the lower caffeine-containing layer was mixed with 10 mL chloroform and placed in a quartz cuvette. Absorbance was measured at 274 nm, and the caffeine content g/100 g IP extract was calculated according to Equation (1).

(1) $$Caffeine\left(\frac{g}{100\;g}\right)=(\frac{\left(A-b\right)\times v}{a\times P\times 1000})$$

where

A = Absorbance at 274 nm; 

v = final solution volume (100 mL);

b = linear coefficient of the calibration curve; 

a = angular coefficient of the calibration curve; 

P = sample mass (g).

#### 2.3.3. Flavonoid Content, Chlorophyll Content, and Antioxidant Activity

The flavonoid content was determined spectrophotometrically [29], using quercetin as a standard reference and distilled water as the solvent for all samples. The results were obtained after calculating and correcting the extract or herb mass and solvent volume. Chlorophyll extraction was performed using 0.5 g of IP or concentrated IP extract in 20 mL of 80% acetone, using a porcelain crucible and pistil for maceration [30].

The antioxidant activity was assessed by 2,2-azino-bis-3-ethylbenzothiazoline-6-sulfonic acid (ABTS) and 1,1′-diphenyl-2-picrylhydrazyl (DPPH) methods. The DPPH test was adapted from the method proposed by Baliyan et al. (2022) [31]. It was performed by adding 50 mg of the dry IP extract in 5 mL of the DPPH solution (24 milligrams of DPPH were dissolved in 100 mL of methanol to make the stock solution). Pure DPPH solution was used as the control. After the reaction, the absorbance at 517 nm was read by UV/Visible spectrophotometer (Jasco, V-630 Deutschland, Pfungstadt, Germany). The concentration necessary for inhibition at 50% (IC_50_) was used to calculate the inhibition percentage and values were expressed in g of DPPH per g of extract.

For the ABTS radical test carried out according to Rufino et al. [32], first, a potassium persulfate solution was prepared after preparing the ABTS radical. Samples (200 μL) of the formed radical were pipetted and diluted in ethanol (PA, 99%) up to an absorbance of 0.7, determined at 734 nm in a spectrophotometer (Hitachi UV spectrophotometer, U-1800). Next, an aliquot of 980 μL of the diluted radical was added to 20 μL of the sample, followed by stirring for a few seconds, and after 7 min, another absorbance measurement (A754 = Af) was performed. For this test, Trolox was used as standard (15 μM = 0.13209 g/500 mL). The result was calculated with the radical inhibition percentage expressed by μmol TEAC/g (Trolox equivalent antioxidant capacity).

#### 2.3.4. Gas Chromatography

To perform the gas chromatography analysis, the sample of *Ilex paraguariensis* extract (500 µL) was mixed with approximately 300 mg of anhydrous sodium chloride salt until saturation, followed by intense mixing using a vortex mixer for 15–20 s. Then, 500 µL of ethyl acetate was added to the mixture and mixed for 1 min using a vortex mixer. After this, the mixtures were centrifuged for 2 min at 1000× *g* to separate the phases, and the non-aqueous phase was used in GC-MS. The sample was analyzed using a gas chromatograph (GC-2010 Plus, Shimadzu, Kyoto, Japan) coupled to a mass spectrometer (GC-MS Ultra 2010, Shimadzu, Kyoto, Japan) using a DB-5 fused silica capillary (J and W, Folsom, CA, USA) with 5% of phenyl dimethylpolysiloxane on capillary fused silica (30 m long × 0.25 mm internal diameter × 0.25 μm film thickness). To perform the analysis, 1 µL of the sample was injected into the GC under split mode at a 100:1 split ratio under a constant flow of 48.851 mL min^−1^ on the column. The temperature of the injection was kept at 180 °C. The GC oven temperature was initially held at 50 °C for 1 min and then raised to 200 °C at 40 °C min^−1^. The total running time for this method was 4.75 min. The interface and detector temperatures were 210 °C and 150 °C, respectively. The MS detector was turned off between 2.03 and 2.21 min to offload the ethyl acetate peak. The retention time and mass spectra of ethanol and methanol were used to identify the compounds.

### 2.4. Definition of Experimental Planning 

The effect of applying different concentrations of IP extract (X1) and sorbitol (X2) on film production was studied through a central rotational composite design (CCRD), containing four central and four axial points [33]. Table 1 shows the adopted design. The α value (α is the distance between each axial point (also called the star point) and the center) was calculated by the ranges of variation between the lower and upper limits of each independent variable. It was established according to the data obtained in pre-tests as a function of the number of independent variables (n = 2) through Equation (2).

∝ = ∜(2^n^) = ∜(2^2^) = 1.41 (2)

The significance of regression and lack of adjustment was 95% confidence (*p* ≤ 0.05) for the planning studied.

As a reference for the results discussion, the following statements were used [33]:

- For the F test, the value of Calculated F divided by Tabulated F (FC/FT) for regression should be greater than one so that the mathematical model is satisfactory and predictive, and the FC/FT for misadjustment should be less than one, thus there are no significant selection errors in the analyses. Tabulated F was defined by Rodrigues and Iemma’s book (percentage points of F distribution, 5%) [33].
- The estimated effect indicates how much each factor influences the responses studied. The higher its value, the greater the influence. A positive effect indicates that it goes from a minimum to a maximum variable value and a maximum response. The negative effect indicates the opposite.

### 2.5. Production of Edible Films 

The films were developed using the casting method, with a fixed pectin concentration (3% *w*/*v*); the extract and the plasticizer (sorbitol) concentration varied according to the above experimental design (Table 1). Initially, the IP extract was added to distilled water and mechanically stirred (Fisatom 713DS, São Paulo, Brazil) for 3 min. Then, the plasticizer was added and stirred for over 3 min, with both processes carried out at room temperature. Next, the pectin was added and mechanically stirred in this solution for 20 min at 35 °C. After that, the filmogenic solution was shaken on ultrasound equipment for 20 min at room temperature to completely dissolve the components and remove the bubbles resulting from stirring. Finally, approximately 50 mL was added to plastic plates 16 cm in diameter and dried in a forced-air oven at 35 °C for approximately 18 h. Before the analysis, all edible films were stored in desiccators containing magnesium nitrate (Mg(NO_3_)_2_), controlling the relative humidity of 58% at room temperature for at least 48 h.

### 2.6. Characterization of Edible Films 

A summary of the analysis performed on films and dried extract of *Ilex paraguariensis* is available in Table 2 to clarify the characterizations made for each sample. The analysis descriptions are presented below.

#### 2.6.1. Thickness, Water Vapor Permeability (WVP), and Solubility in Water 

The thickness of each film was measured using a digital micrometer (Mitutoyo). The thickness measurements were carried out at five different points of each sample, and these values were used to calculate the mean and standard deviation. Water vapor permeability was determined gravimetrically following Martelli et al. (2006) [34]. A capsule with a permeation area of 0.005 m^2^ containing anhydrous calcium chloride and an analytical balance with a resolution of 10^−4^ g (Mettler Toledo, Columbus, OH, USA) were used to determine WVP. The test was performed at 25 °C and 75% RH in an air conditioning chamber. The calcium chloride was previously dried in a ventilated oven at 140 °C for 24 h. These cells were placed in a hermetic chamber with anhydrous calcium chloride desiccant, which was placed in a constant temperature (35 °C) oven. In this way, the weight variation of each cell over time due to an RH gradient of 0–75% was determined. The cells were weighed every 30 min, using a semi-analytic scale of 12 h, and a linear relation between the quantity of water transferred per unit of area and time was obtained. The WVP was calculated by applying Equation (3).

(3) $$WVP=\frac{G}{t}\frac{e}{A\;\rho_{s}(RH_{1}-RH_{2})}$$

where WVP is in g mm/m^2^ day KPa; e is the average thickness of the pectin films (mm); A is the permeation area (0.005 m^2^); RH_1_ is the chamber RH; RH_2_ is the RH inside the cells; ρs is the vapor saturation pressure in the oven (KPa); and the term G/t (g water/day) was calculated by linear regression from the weight variation over time data, in a steady state.

The total water solubility of the films was evaluated according to Fakhouri et al. (2018) [35].

#### 2.6.2. Color Parameters, Fluorescence, Photodegradation, and UV/Vis Light Barrier Property

The color analysis was performed by reading directly on the surface of the films on a white background, using the digital colorimeter CR 400 (Konica Minolta). The parameters L*, chroma a*, and chroma b* were determined in the films with and without extract, and the color difference (ΔE*) was calculated using standard data from test 5 (without extract).

For fluorescence analysis, a film containing 15% (*w*/*w*) IP extract was prepared, in order to better observe the phenomena. Fluorescence measurements were performed using a portable spectrofluorometer composed of two lasers, one operating at 405 nm and the other at 532 nm, a monochromator (USB 2000 FL/Ocean Optics, Santa Clara, CA, USA), a Y-type fiber, and a laptop computer. In this work, only the laser of 405 nm was used as excitation. All the samples were placed at a fixed distance from the laser beam. The fluorescence spectra were determined using the Spectra Suit^®^ program (Santa Clara, CA, USA), for 5 h to verify if light radiation changes the natural fluorescence of the films. The UV/Vis technique was used to determine the optical absorbance of the films in the wavelength between 200 nm and 800 nm in the spectrophotometer Varian Carry Eclipse 50 (Santa Clara, CA, USA).

#### 2.6.3. Thermogravimetry (TGA)

The TGA measurements were performed at the Faculty of Exact Science and Technology Lab Facility at the Federal University of Grande Dourados, Dourados, Mato Grosso do Sul State, Brazil. 

The thermogravimetric analyses (TGA) were performed on a Shimadzu (Kyoto, Japan) TGA-50 high-sensitivity thermobalance, coupled with a microprocessor for programming heating and cooling modes, interfaced with the TA50-WSI data station system. The heating temperature was 15–700 °C under a nitrogen atmosphere with a heating rate of 10 °C/min. The sample mass used for each film during the analysis was 3.0 mg, and the samples were cut in 2 mm^2^ sizes.

### 2.7. Statistical Analysis 

The tests were performed randomly, and the statistical analysis results were obtained using Statistica^®^ 7.0. The results were verified by analysis of variance (ANOVA) using the F test.

## 3. Results

### 3.1. Ilex paraguariensis Extract 

**Figure 1:**
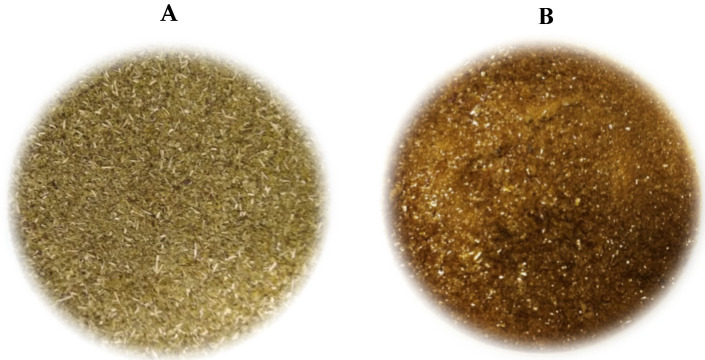
Visual aspect of the commercial “extra strong” *Illex paraguaiesis* (**A**) and extract prepared in methanol/water (1:4) from it (**B**).

The yield obtained from a dried hydrophilic extract was approximately 34.7 g/100 g of IP, and according to chromatography analysis, no residues of methanol were present in the extract, which showed a high application potential. Visually, the extract had a different color and appearance compared to IP, showing a bright aspect, as seen in Figure 1.

Considering the color parameters of IP and IP dried extract, measured instrumentally, the chroma b* presented a slight difference between the samples, including these values within the deviation of the analysis. IP presented a higher value in parameter L* (Table 3) when compared to the dried extract; the IP was lighter. However, it is noteworthy that such a parameter determines the clarity (white and black intensity) and not the reflectance, which was not changed by the brightness of the extract.

Table 4 shows the concentrations found in commercial IP and dried extract prepared to analyze phenolic compounds, caffeine, flavonoids, chlorophyll, DPPH, and ABTS. 

The results obtained in determining the total phenolic content by the Folin–Ciocalteu method were expressed as mg of gallic acid equivalents (mg GAE) per g of extract or g of IP. These results were obtained after making the corrections with the extract or IP mass and solvent volume, and it shows that the IP extract had a higher phenolic compound concentration than the pure IP. 

Caffeine determination results (Table 4) were calculated through the standard curve, where a straight-line equation with linear regression of 0.99 was obtained. The extract has a caffeine concentration approximately 5.4 times higher than pure IP.

The extract presented a higher content of flavonoids than IP.

As seen in Table 4, DPPH and ABTS results show that the extract has a higher antioxidant activity than pure IP.

Chlorophyll analyses were performed to find the concentration of chlorophylls *a* and *b* as can be seen in Table 4. Chlorophyll *b* concentration did not differ between the concentrate extract and IP, showing no chlorophyll loss during processing. However, a small loss of chlorophyll *a* occurred, influencing the total chlorophyll concentration.

**Figure 2:**
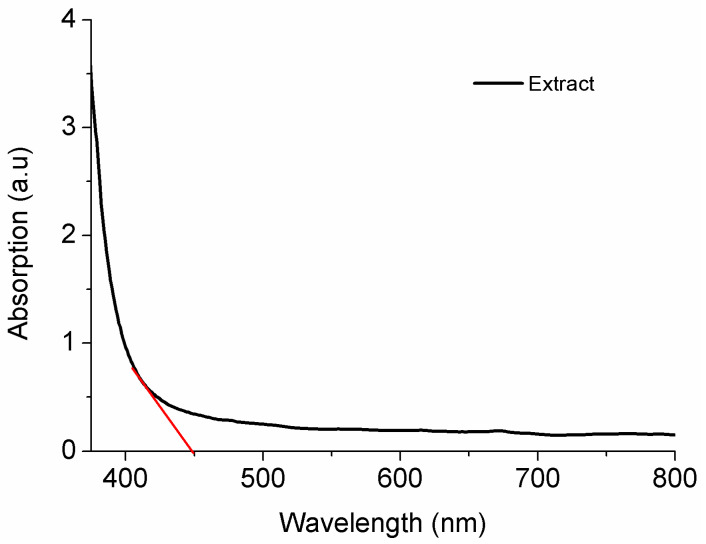
Transmittance profile of *Ilex paraguariensis* extract in the UV and visible regions.

**Figure 3:**
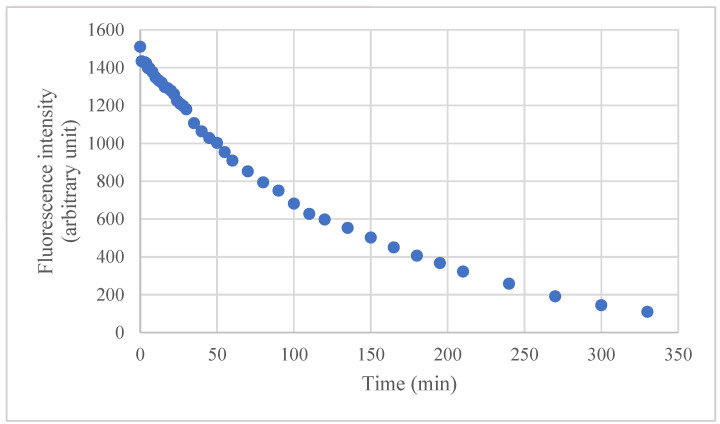
Degradation curve of dried extract exposed to UV light for 335 min at 405 nm.

Regarding the physical analysis of the concentrated extract, Figure 2 shows the optical profile of the extract studied by UV/Vis spectroscopy for the UV (365–800 nm) region, and Figure 3 shows the degradation curve of the extract exposed to UV light for 335 min at 678 nm. 

The transmittance of the solution in the visible region (400–700 nm) is close or equal to 100% [36]. From optical absorbance (A) measurements, the optical absorption coefficient was determined by the Beer Lambert’s formula $a=2.303\left(\frac{A}{d}\right)$, where d is the thickness of the sample.

### 3.2. Films

**Figure 4:**
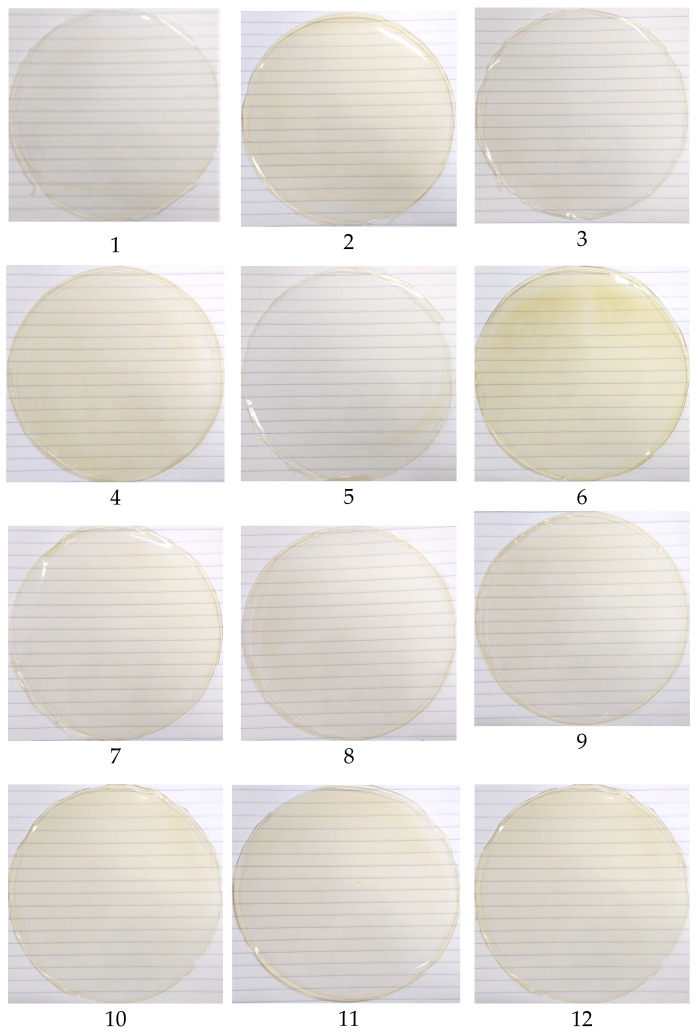
Edible and biodegradable films developed. The numbers (1 to 12) correspond to the test according to the experimental design used.

The films resulting from the conditions defined by the experimental design can be seen in Figure 4. The photos were obtained on a striped background for better visualization of the films’ transparency. All the films are transparent, glossy, and flexible, with minor differences in their thickness, as a consequence of sorbitol concentration (Table 5).

#### 3.2.1. Thickness, Water Vapor Permeability, and Solubility in Water

**Figure 5:**
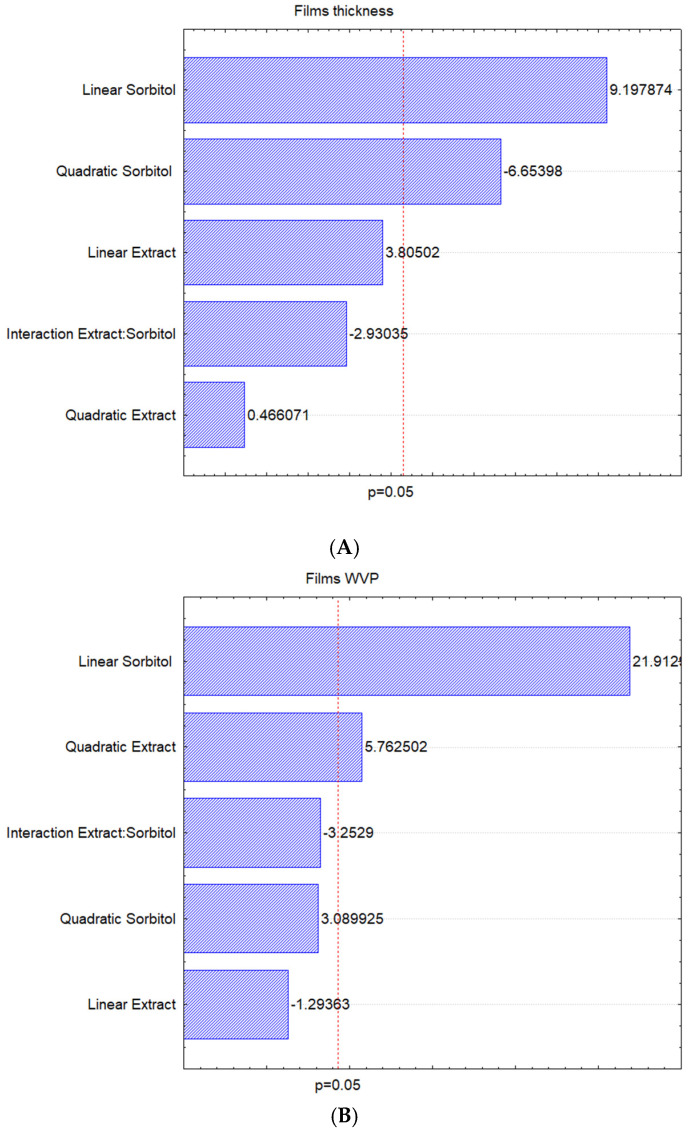

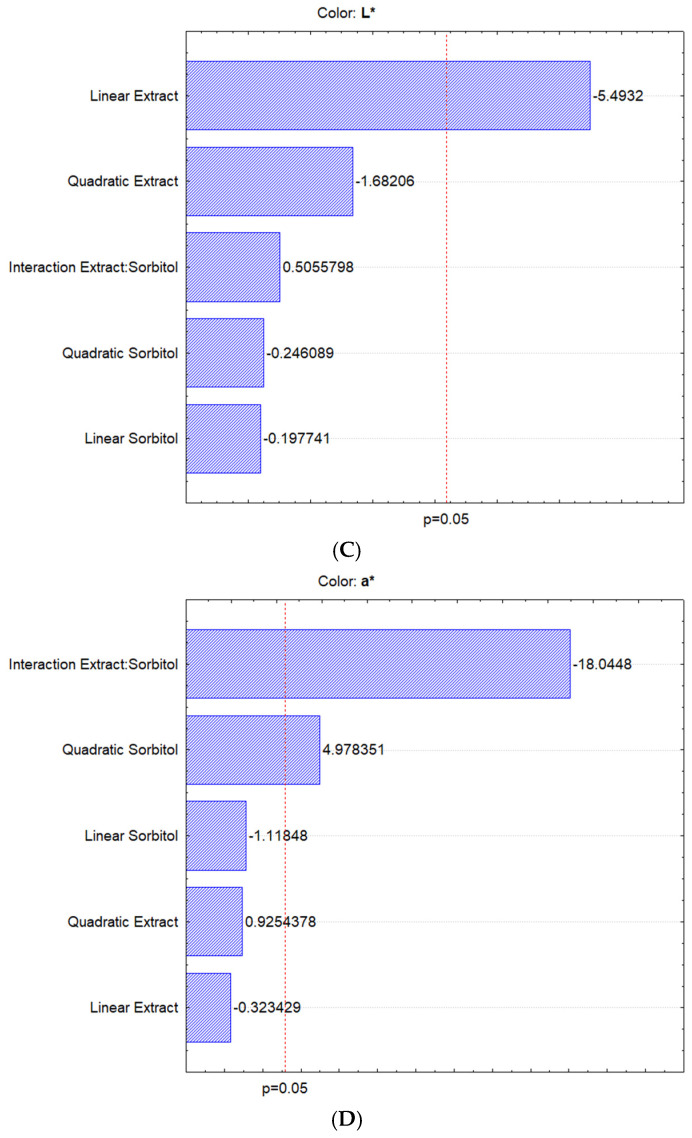

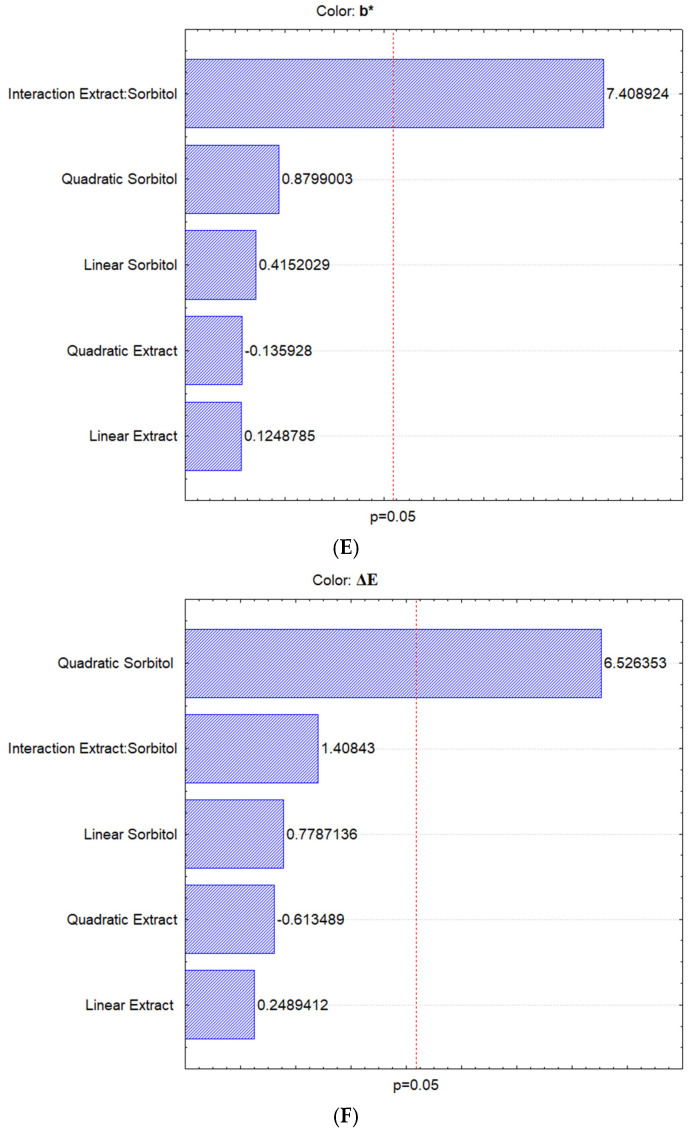

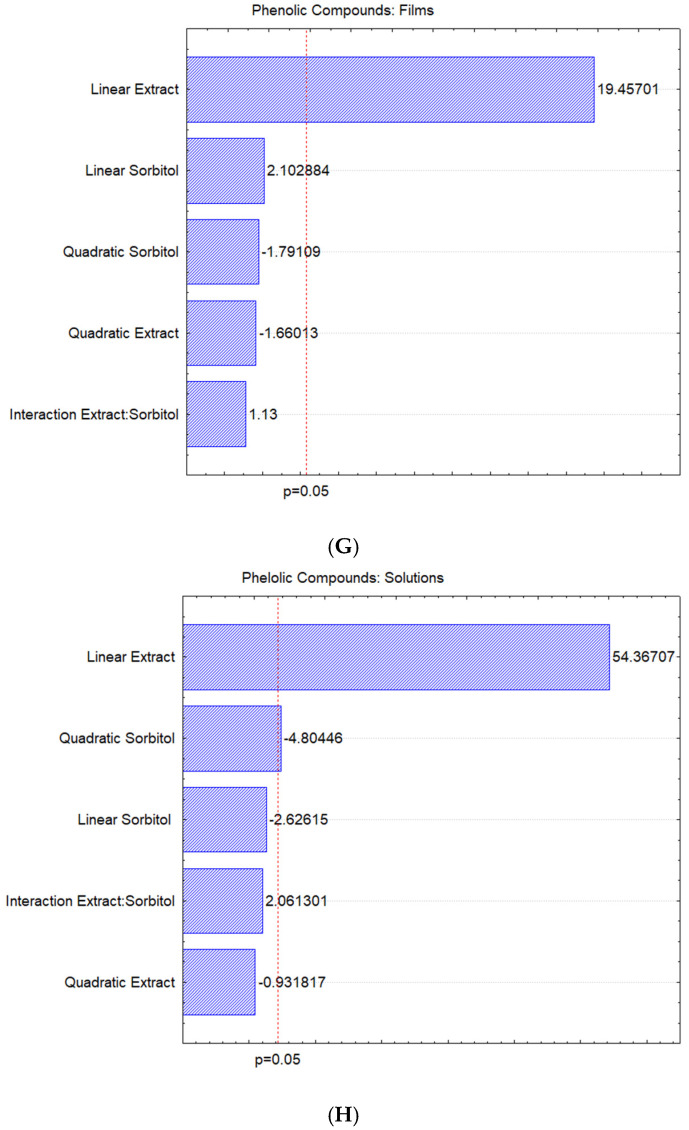
Pareto graphs of the effects from sorbitol and IP extract concentrations in the analysis: (**A**) thickness; (**B**) WVP; color parameters (**C**) L, (**D**) a*, (**E**) b*, and ΔE (**F**); and total phenolic content of the (**G**) films and (**H**) solutions.

Figure 5 shows the Pareto graphs obtained through the statistical analyses applied to thickness, WVP, solubility, color parameters, and total phenolic content to films and solutions data, and Table 5 presents the results for the same analyses described before, according to the adopted experimental design.

When performing the experimental design, only sorbitol had a significant influence on the thickness of the films, as seen in Figure 5. However, to improve the coefficient of determination of the mathematical model (Equation (2)), all factors were maintained. Plasticizers can significantly increase the thickness of a film because plasticizers disrupt and restructure the polymer chain networks, creating more free volumes [37,38].

Applying the analysis of variance, the relationship between FC/FT for the regression was less than 1, so the mathematical model is not satisfactory. However, the lack of adjustment was less than 1; thus, the analysis has no significant variation errors. It is assumed that sorbitol negative (Quadratic Sorbitol) and positive (Linear Sorbitol) influence film thickness. As the mathematical model was not satisfactory from the analysis of variance and the coefficient of determination was 0.79, showing that the mathematical model (Equation (4)) predicts 79.02% of the data variation, it was decided not to proceed with the statistical analysis due to the unfeasibility of the model.

FT = 0.087 + 0.003E + 0.000410E^2^ + 0.007S − 0.006S^2^ − 0.003ES (4)

where

- FT = film thickness (mm);
- E = concentration of extract added to film (g);
- S = concentration of sorbitol added to film (g).

**Figure 6:**
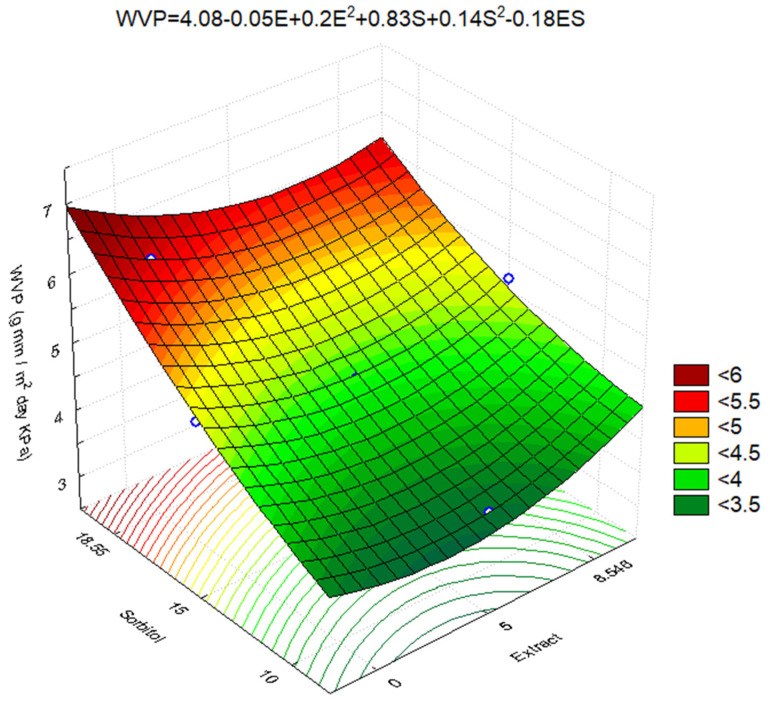
Water vapor permeability surface graph of films developed according to experimental design.

Among the variables, Linear Sorbitol stood out and had the most significant influence on the permeability of the films (Figure 5), showing a positive influence. The Linear Sorbitol is followed by the Quadratic Extract, which also has a positive influence on the response (Figure 5). The other factors did not present significant influence but were kept to improve the model fit. The generated mathematical model is represented in Figure 6.

Where

WVP = film permeability (g mm/m^2^ day kPa);

E = concentration of extract added to film (g);

S = concentration of sorbitol added to film (g).

From the applied analysis of variance, the FC/FT of regression presented values above 1, so the mathematical model (Equation (4)) is considered satisfactory (93.66%). There was no lack of adjustment in the analysis (FC/FT < 1), so the error proved uninfluenced. The surface plot of the reduced response of the experimental design for water vapor permeability is expressed in Figure 6. 

For the film to show a lower WVP, the addition of extract must be within 1.5% and 6.8%, and the addition of sorbitol should be equal to the lowest concentration tested by this study, which was 10%. Although WVP decreases as sorbitol is removed from the film, reducing this variable below 10% is not pleasant because the plasticizer gives the film flexibility.

All analyzed films were 100% soluble, so there was no significant variation in this response in the experimental design. This result was expected since the IP extract was hygroscopic, and when sorbitol and pectin were applied, this behavior did not differ.

#### 3.2.2. Color Parameters 

**Figure 7:**
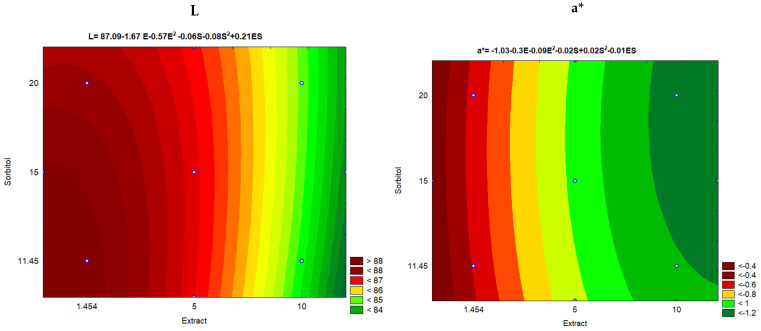

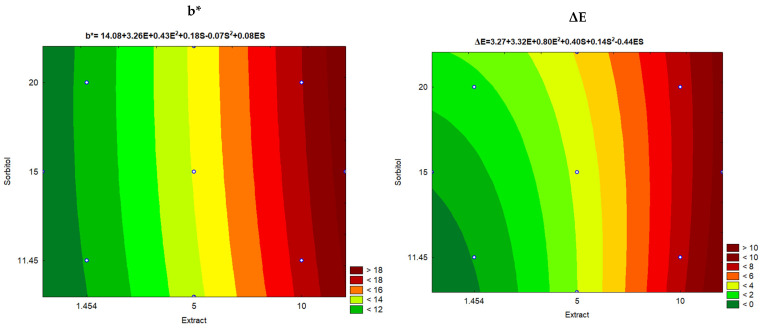
Contour curves for color parameter responses (L, a*, b*, and ΔE).

Overall, adding IP extract had little influence on the color of films, which can be visually observed in Figure 7. In all responses, the most significant variable was the Linear Extract. This effect was negative for L and a*, and positive for b* and ΔE. Only the a* response had one more significant variable that had a positive effect. The positive effect for the b* parameter indicates a tendency to yellow coloration, as observed in the edible films (Figure 7), which was expected due to the characteristic coloration of the extract.

The mathematical models representing the responses of L, a*, b*, and ΔE are expressed in Figure 7. 

Where

- L = film brightness;
- a* = response from a* (green to red variation);
- b* = b* response (range from blue to yellow);
- E = extract concentration added to the film;
- S = concentration of sorbitol added to the film.

From the analysis of variance, all mathematical models were considered satisfactory except for a*. Although the lack of adjustment of the response to a* was significant, the value was close to one (1.4), with little interference with the generated mathematical model. All coefficients of determination were above 0.8, thus explaining more than 80% of the results.

#### 3.2.3. Total Phenolic Content 

**Figure 8:**
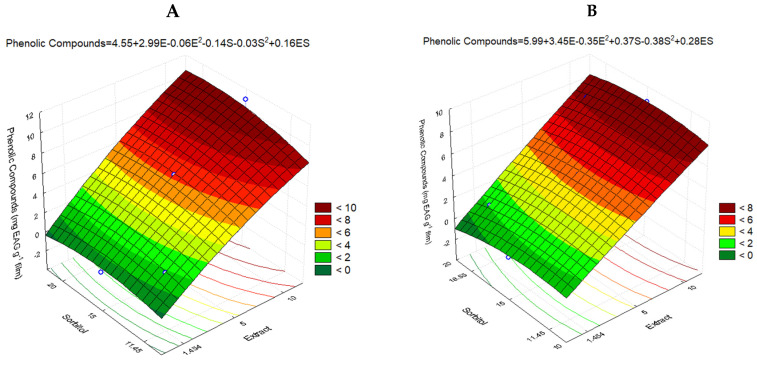
Fitted surface of total phenolic content from (**A**) films and (**B**) solutions.

The non-significant effect parameters were not discarded, so the determination coefficient (R2) was 0.9773 and 0.9801 for films and solutions, respectively, showing that both models explain at least 97% of the data variation. The mathematical models that represent the total phenolic content in solutions and films are expressed in Figure 8A and 8B, respectively. 

Where

- CFS = solutions of phenolic compounds (mg GAE g^−1^);
- CFF = films of phenolic compounds solutions (mg GAE g^−1^);
- E = extract added to film (g);
- S = sorbitol added to film (g).

The analysis of variance was satisfactory, and the FC/FT of regression for solutions and films were greater than 1, suggesting that both mathematical models are effective and predictive. The lack of adjustment of the films was not significant (FC/FT < 1), and although the lack of adjustment of the solutions was above 1 (1.01), the value was very close and did not interfere with the coefficient of determination or in the data prediction.

The parameter that significantly influenced the concentration of phenolic compounds in films was the Linear Extract, which had a positive influence. In the solutions, Linear Extract and Quadratic Sorbitol had an influence of being Linear Extract positive and Sorbitol Quadratic negative. For the other parameters, neither was significant, and the Linear Extract parameter had the greatest effect on both. 

Figure 8 shows the surface graphics of solutions and films, in which the dark red color represents the most extensive total phenolic content. Within the chosen range (according to experimental design), the total phenolic content is proportional to the addition of IP extract; when the IP extract is added, the total phenolic content increases.

Although not presented as a significant influence factor, Figure 8A shows a slight increase in the concentration of phenolic compounds with the addition of sorbitol.

#### 3.2.4. Fluorescence and Photodegradation of Films 

**Figure 9:**
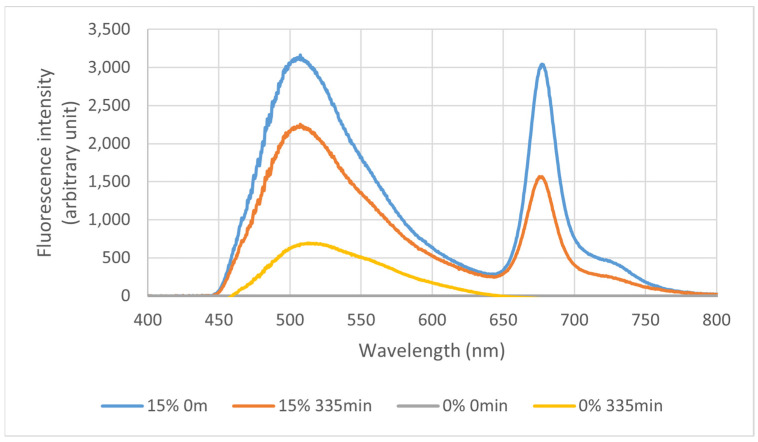
Fluorescence spectra of films with 15% extract (15%) and no extract (0%), both at times 0 and 335 min.

The fluorescence of the samples was measured, and the result can be seen in Figure 9. The films presented two light emission peaks, one larger for pectin and sorbitol (between 450 and 600 nm) and one smaller for the extract (640 and 750 nm). The peaks at 625 nm and 725 nm refer to chlorophyll *a* and *b* from the extract. As seen, the fluorescence intensity decreases with the increase in exposition time, demonstrating a photodegradation effect.

**Figure 10:**
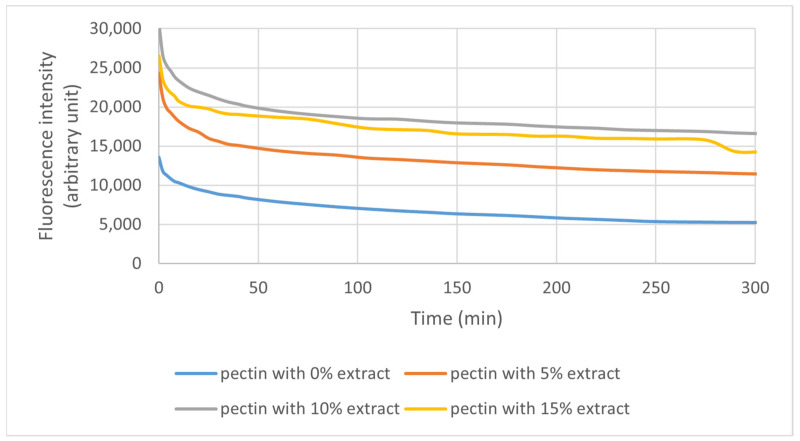
Pectin degradation curves of films exposed to UV light for 335 min at 505 nm.

Figure 10 presents the photodegradation measurements for pure pectin and films containing IP extract. The film thickness standardized the results. For the exposition time of 335 min, pure pectin is more stable than other films. It is possible to see that the increase in extract amount decreases the film stability.

**Figure 11:**
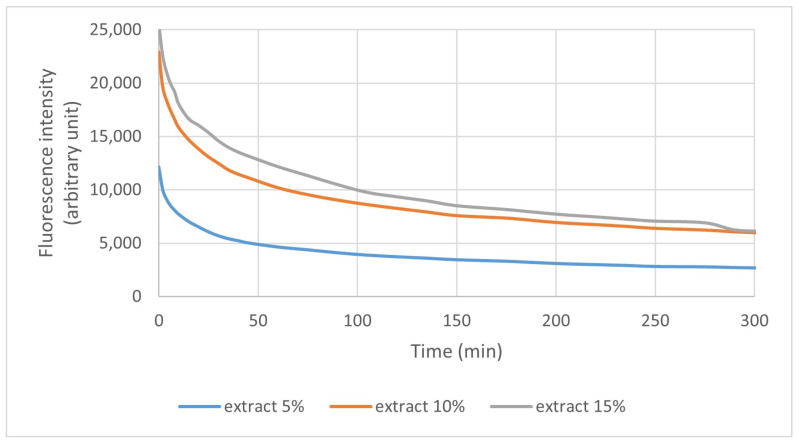
Degradation curves of extracts present in films in the same concentration exposed to UV light for 335 min, at 678 nm.

Figure 11 shows the fluorescence peaks of the extract in the films. As expected, the highest fluorescence intensities for the extracts in the films was with 15% extract, followed by the 10% and 5% formulation. The formulation without extract did not show this peak, as seen in Figure 9.

#### 3.2.5. Thermogravimetry (TGA) 

**Figure 12:**
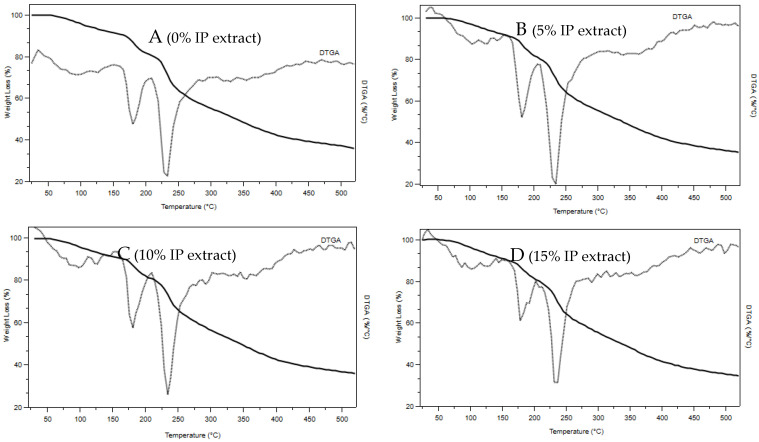
Thermogravimetry (TGA) and derivative (DTGA) traces of pectin films with different IP concentrations: (**A**) pectin; (**B**) pectin with 5% IP extract; (**C**) pectin with 10% IP extract; (**D**) pectin with 15% IP extract.

The TGA technique was applied to characterize the thermal transitions of pectin-based films and extracts (Figure 12). The incorporation of IP extract shows no influence on thermal degradation, as shown in Figure 12; there is no significant difference between the tested films. No significant mass losses were observed on the TGA curve. The first mass loss, from room temperature up to 200 °C, can be attributed to the free water in the films [39].

## 4. Discussion

### 4.1. Ilex paraguariensis Extract

Regarding the color parameters, a* showed a more significant difference between the tested samples, where IP tended to be green (−a* indicates green), and the extract tended to be red (+a* indicates red). However, both values were close to zero, showing low saturation. The color difference of the samples (ΔE) was small; considering how much closer to zero the value of this variable was, the smaller the difference; the extract showed characteristics close to the commercial IP. Other authors found different values of L, a*, and b* for IP; some examples can be seen in Table 6.

The experimental color results for IP in this study are close to the values found in the literature. However, as seen in Table 6, these values vary from one cultivar to another or are due to the selection process or drying. Thus, the color difference between the matt herb and the dry extract is a little expressive.

In a study using the Folin–Ciocalteu method, authors obtained values of phenolic compounds between 49.84 and 110.4 mg g^−1^ for IP using different solvents [43]. Among them, methanol had a total phenolic content 107.43 mg g^-1^, which does not differ from the best extraction solvent (water by decoction).

The flavonoid compounds have beneficial effects, such as antioxidant, anti-inflammatory, antiviral, or antiallergic action; these characteristics allow it to be used in food technology as a natural preservative [44]. Ribani [27], through the analysis of high-performance liquid chromatography (HPLC), found flavonoid values between 2.59 and 4.94 mg QE g^−1^ for IP, values lower than those obtained in this study (Table 4). However, in their study on phenolic substances, flavonoids, and antioxidant capacity in plants under different soil cover and shading, Ferrera et al. [45] found values for flavonoids between 30.91 and 286.04 mg catechin equivalent per L of extract; this denotes an intense change in this class of compound because of edaphoclimatic changes in production.

Regarding DPPH, the obtained result in this study agrees with that found by Serafim (2013) [46], who presented IC_50_ values between 2.77 and 3.79 mg mL^−1^ for dry leaves of IP and with that found by Zanella Pinto [47] who found IC_50_ values of 2.33 to 2.78 mL extract/mg of coarse-ground IP [46]. The DPPH data indicate that the dry extract has an IC_50_ value of 0.81 mg mL^−1^, significantly lower than that of commercial yerba mate, which is 2.74 mg mL^−1^ (Table 4), suggesting that the dry extract has a superior antioxidant capacity, being more effective in neutralizing free radicals. This superiority can be attributed to a higher concentration of phenolic compounds and other antioxidants in the dry extract, known for their beneficial action in mitigating oxidative stress. According to Feihrmann et al. (2022), yerba mate extract has been used in some foods as a substitute for synthetic antioxidants [48].

Complementing this analysis, the results of the ABTS assay corroborate the trend observed for DPPH. The dry extract presents a superior antioxidant activity to commercial yerba mate, reinforcing the evidence that the dry extract is more effective in capturing free radicals.

The ABTS antioxidant capacity for IP was lower than that found in the literature (148.47 μM Trolox g^−1^), where Vieira et al. (2009) obtained results between 240.33 and 272.37 μM Trolox g^−1^ for IP powder [49,50]. However, it is noteworthy that the raw material used strongly influences the compounds present in IP. These compounds vary from one cultivar to another, subject to planting, harvesting, and industrialization conditions. However, the extract was significantly more concentrated when compared to IP, as seen in Table 4, so the applied methodology was efficient in concentrating the antioxidant compounds originally present in IP.

From the caffeine results, the IP extract showed to be a promising additive for edible films, where even in small concentrations, adds this powerful central nervous system stimulant to the film. Moreover, caffeine works by blocking adenosine, the neurotransmitter of sleep. When ingested in an appropriate concentration, caffeine decreases drowsiness, apathy, and fatigue, favoring the consumer’s intellectual activity, and increasing attention span, concentration, and memory [49].

Braghini et al. [51] found caffeine concentrations from 0.82 to 2.45% in IP, a value lower than that found in this study (4.77%). This can be explained by caffeine varying from plant to plant depending on product origin, environmental conditions, climatic factors, crop treatments, plant genetic variety, pest occurrences, growing conditions, cultivation, and pruning techniques. Even seedling shading interferes with the concentration of caffeine in the IP, as shown by Coelho et al. [52], who studied plants under different degrees of shade in seedlings and observed a variation from 2.31 to 14.31 mg g^−1^ caffeine.

There are four types of chlorophyll: *a*, *b*, *c*, and *d*. Chlorophyll *a* is the most common type found in almost all photosynthetic organisms, and type *b* has a higher concentration in shaded plants. Chlorophylls *c* and *d* are uncommon in traditional plants; thus, chlorophylls *a* and *b* were quantified. Chlorophyll reference values for commercial IP have not been found to compare. However, chlorophyll characteristics are attributed to good antioxidant, antimutagenic, and chemo-preventive activity; these characteristics could be attributed to IP extract due to the presence of this compound [53].

In the UV region, the extract (Figure 2) shows some potential as a film additive because it can act as a barrier to most ultraviolet rays. This is relevant for food preservation once exposure to light can degrade color and taste, and exposure to light accelerates oxidation reactions and, as far as possible, their direct incidence on oxidation-susceptible foods should be avoided [54]. Chlorophyll has a complex structure; it is a chlorin, porphyrin derivative, a cyclic tetrapyrrole with an isocyclic cyclopentanone ring containing double blonds [55], which can absorb and reflect light in different wavelengths and thus contribute to reducing the light transmittance to food. 

In fluorescence spectroscopy, the emitted light (fluorescence) is proportional to the concentration of the analyzed compound. Thus, because of the degradation by UV light, the emission light tends to decrease as a function of the exposure time. Initially, the extract showed a fluorescence peak of 1511 a.u. at 0 min, and degraded to 109 a.u. at 330 min. The curve obtained through this analysis is also seen in the dried extract in the films, which is discussed below. As the evaluated sample was diluted in water, there were no changes in the color or behavior of the extract. Yet, an intense decrease in light emission can be observed during exposure time, showing the degradation of the compounds originally present in the IP. However, it is essential to detach the high stability of these compounds, in which, during the analysis, the degradation is slow, considering the laser intensity (±10 mW) to which the sample was submitted, thus showing good light stability of the compounds in the sample.

### 4.2. Films

#### 4.2.1. Thickness, Water Vapor Permeability, and Solubility in Water

In this study, the thickness of the samples is similar to those observed in the literature for pectin-based films. Maftoonazad et al. (2007), who developed pectin films without the addition of plasticizer, observed a thickness of 0.06 ± 0.01 mm [56]; this value corresponds to tests 1 and 7, with the lowest plasticizer concentration. This test clarifies that sorbitol greatly influences the film thickness, Sorbitol Linear positively influences, and Quadratic Sorbitol negatively influences it. 

Regarding WVP, Krochta and De Mulder-Johnston (1997) rated the films according to a range of values for WVP, classifying the films as unsatisfactory (10 to 100 g mm/m^2^ Day kPa), moderate (0.10 to 10 g mm/m^2^ day kPa), or good (0.01 to 0.10 g mm/m^2^ day kPa) [57]. According to this classification, all developed films are moderated regarding the water vapor barrier, where the WVPs of the developed films were between 3.35 and 5.78 g mm/m^2^ day KPa. As sorbitol concentration increased, the film permeability also increased, and the sorbitol concentration caused an increase in the equilibrium moisture content of films [58,59]. Martelli et al. (2006) concluded that this behavior is expected since films with a higher concentration of plasticizer absorb more moisture at a given water activity because plasticizer molecules provide more active sites to bind water molecule hydroxyl groups [58,60]. This behavior corroborates with the data found in the literature, as when sorbitol is added to the film, an interaction with pectin molecules occurs, giving the film flexibility, hydrophilicity, and increasing WVP [61].

In his study on alginate incorporation in cross-linked pectin films, for pectin-only films and varying glycerol concentration, Bierhalz (2010), found that the values of WVP were between 2.45 and 6.01 g mm/m^2^ day KPa, where the glycerol concentration ranged from 1 to 7%, this being the highest WVP value for the film with the highest glycerol concentration. These data agree with this study, where the identified WVP range is similar, and by increasing the plasticizer (glycerol or sorbitol) concentration, the permeability increases [62].

The films were 100% soluble. According to Shih (1996), polysaccharides are highly hygroscopic and disintegrate rapidly in water, which is in accordance with the result obtained in this study. Therefore, due to this characteristic, the films are biodegradable and fit into the “green technology”, and they help to preserve the environment [63].

#### 4.2.2. Color Parameters

Overall, the films were transparent, bright (L ≥ 82), and yellowish green (a* negative and b* positive). Sorbitol had no influence on the color of the films (Figure 7). There is no limitation on a film’s color, as the color will depend on future applications. However, the range in which the added extract film differs less from the “standard film” (test 5, without extract) is between 0.04% and 3.23% of extract in the film.

#### 4.2.3. Phenolic Compound

The total phenolic content in films is proportional to the addition of IP extract; when the IP extract was added, the total phenolic content increased. However, considering that the purpose was to develop an edible film, a higher concentration of extract (over 10%) would make the film taste bitter and not palatable.

A slight increase in the phenolic content was observed with the addition of sorbitol. This infers that sorbitol helps stabilize these compounds and makes them less susceptible to degradation. Thus, as the point of highest concentration of phenolic content in films and solutions, we can select the positive axial points, where the highest concentrations of extract and sorbitol are. The optimum concentration of the variables for the elaboration of the films, in terms of phenolic content, is 10% extract and 20% sorbitol.

Farias et al. (2021) prepared sodium alginate films with yerba mate extract and investigated the release of total phenolic compounds (TPC) and chlorogenic acid (CA) in different food simulants (Simulants A = water, B = 10% ethanol, C = 3% acetic acid, and D = isooctane PA) [64]. The release of TPC in the different simulants showed diffusivities of the same order of magnitude. Still, the films with 25% of yerba mate extract displayed higher release rates in aqueous media, indicating that they can be used as active packages in acidic or nonacid hydrophilic foods, acting as natural antioxidants, avoiding or retarding the oxidative reactions of the food’s components [64]. Rezzani et al. (2024) produced films using water kefir grains as a polymeric matrix and IP extract, the films had remarkable antioxidant activity, with a maximal radical scavenging activity (%RSA) of 98  ±  2 and 71  ±  2 measured by ABTS and DPPH assays, respectively [65].

#### 4.2.4. Fluorescence and Photodegradation of Films

As the concentration of pectin and sorbitol added to the films did not vary, the difference in fluorescence intensity presented can be attributed to the presence of the extract. Before the assay, the films were stored for 2 days, with a slight incidence of light, which may have caused the degradation observed in the analysis. Considering that the extract maintains pectin and sorbitol with greater stability, the films added with the extract were initially less degraded at the end of the study.

The 10% extract pectin/sorbitol fluorescence peak curve was more stable than the others, but with a little difference from the 15% extract film, followed by the 5% and last 0% film curve, reaffirming that adding the extract helped stabilize the films. 

The films showed no change in color or behavior because of the small point used; however, an intense decrease in light emission can be observed during exposure, indicating the degradation of the compounds present in the film (pectin, sorbitol, and IP).

#### 4.2.5. Thermogravimetry (TGA) 

In the TGA curves, the degradation between 220 and 250 °C can be related to organic degradation. The films’ most important thermal degradation rate occurs at approximately 235 °C, and the mass loss to this point is 35%. Andrade (2010), in his research on pectin-based films with different sorbitol concentrations, found that the temperature of thermal degradation for such films is 230 °C, according to this study [66]. In the study of Arrieta et al. (2018), it was demonstrated that between 150 °C and 460 °C, IP extract lost almost 60% of the initial mass in two thermal degradation step processes, with maximum degradation temperatures (T_max_) at 215 °C and 315 °C, [67], so in the same range of the pectin matrix. This is why no additional peaks appear in films containing the extract.

## 5. Conclusions

The methodology applied to obtain the extract allowed for the concentration of the compounds originally in *Ilex paraguariensis* to be determined and showed that there are advantages in applying this extract to the films. Even when added in smaller fractions, it adds significant amounts of compounds that contribute to antioxidant and stimulant characteristics of these films, with low visual and olfactory modification due to the possibility of lower insertion of this additive. The optimum concentration of extract and sorbitol in the films was 10% and 15%, respectively. The amount of sorbitol also influenced film thickness. 

The films could carry the compounds originally in *Ilex paraguariensis*. Moreover, they showed efficient characteristics about the water vapor barrier (showing a great variation scale due to the possibility to increase or not increase the sorbitol concentration, between 3.33 and 5.27 g mm/m^2^ day KPa) and UV light barrier, and were resistant to temperature (around 230 °C to degradation), thus being able to replace conventional primary packaging. Additionally, if consumed with food, these films can add a nutritional value to the packaged product, thus making them functional foods.

## Figures and Tables

**Table 1:** Rotational central composite planning matrix 2^2^ used in the development of edible and biodegradable films.

Test	Coded Variables		Real Value Variables	
	X1	X2	X1 (%)	X2 (%)
1	−1.00	−1.00	1.45	11.45
2	+1.00	−1.00	8.55	11.45
3	−1.00	+1.00	1.45	18.55
4	+1.00	+1.00	8.55	18.55
5	−1.41	0.00	0.00	15.00
6	+1.41	0.00	10.00	15.00
7	0.00	−1.41	5.00	10.00
8	0.00	+1.41	5.00	20.00
9	0.00	0.00	5.00	15.00
10	0.00	0.00	5.00	15.00
11	0.00	0.00	5.00	15.00
12	0.00	0.00	5.00	15.00

X1: *Ilex paraguariensis* extract; X2: sorbitol.

**Table 2:** Analyses performed on films and dried extract of *Ilex paraguariensis*.

Analyses	Extract	Films
Total phenolic content	X	X
Caffeine content	X	
Flavonoid content	X	
Chlorophyll content	X	
Antioxidant potential	X	
High-performance liquid chromatography (HPLC)	X	
Packaging and measurement of thickness		X
Water vapor permeability (WVP)		X
Solubility in water		X
Colorimetric	X	X
Fluorescence and photodegradation	X	X *
UV/Vis light barrier property	X	X *
Fourier-transform Infrared spectroscopy (FTIR)		X *
Thermogravimetry (TGA)		X *
Differential exploratory colorimetry (DSC)		X *

* Analyses carried out in the experimental planning part.

**Table 3:** Color parameters obtained for *Ilex paraguariensis* and the dried extract prepared from this.

Parameters	*Ilex paraguariensis*	Extract
L	49.8 ± 0.8 ^a^	45.0 ± 0.7 ^b^
a*	−0.2 ± 0.2 ^b^	4.3 ± 0.3 ^a^
b*	22.4 ± 0.8 ^a^	22.2 ± 0.4 ^a^
ΔE	-	6.4 ± 0.6

Means followed by identical letters on the same line do not differ from each other, according to the Tukey test, at a 5% significance level.

**Table 4:** Concentrations of phenolic compounds, caffeine, flavonoids, chlorophyll, ABTS, and DPPH determined in commercial *Ilex paraguariensis* and dried extract from this.

Analyses		*Ilex paraguariensis*	Extract
Phenolic compounds (mg GAE g^−1^)		77.74 ± 0.68 ^b^	205.72 ± 0.69 ^a^
Caffeine (g 100 g^−1^)		4.77 ± 0.88 ^b^	25.91 ± 4.71 ^a^
Flavonoids (mg QE g^−1^)		44.43 ± 2.64 ^b^	201.94 ± 37.83 ^a^
DPPH (IC_50_: mg mL^−1^)		2.74 ± 0.01 ^a^	0.81 ± 0.00 ^b^
ABTS (μM Trolox g^−1^)		148.47 ± 1.76 ^b^	287.18 ± 0.00 ^a^
Chlorophyll (mg mL^−1^)	Type a	5.58 ± 0.00 ^a^	4.04 ± 0.00 ^b^
	Type b	5.07 ± 0.00 ^a^	5.08 ± 0.00 ^a^
	Total	10.65 ± 0.00 ^a^	9.12 ± 0.00 ^b^

Means followed by identical letters on the same line do not differ from each other, according to the Tukey test, at a 5% significance level.

**Table 5:** Results obtained in the analyses of thickness, WVP, color (L, a*, b*, and ΔE), and total phenolic content.

Test	Extract (%)	Sorbitol (%)	Thickness (mm)	$\mathrm{WVP}\;(\frac{gmm}{m^{2}dayKPa})$	Color				Total Phenolic Content (mg GAE g^−1^)	
					L*	a*	b*	ΔE	Solutions	Films
1	1.45	11.45	0.067 ± 0.01	3.35 ± 0.08	88.09 ± 1.11	−0.48 ± 0.04	10.77 ± 1.80	0.41 ± 0.77	1.95 ± 0.36	1.27 ± 0.25
2	8.55	11.45	0.087 ± 0.00	3.46 ± 0.09	84.99 ± 1.21	−1.26 ± 0.04	16.68 ± 0.99	6.53 ± 1.18	7.41 ± 0.57	6.77 ± 0.22
3	1.45	18.55	0.088± 0.00	5.78 ± 0.21	88.39 ± 0.95	−0.55 ± 0.04	9.93 ± 1.26	1.18 ± 0.59	2.20 ± 0.23	1.46 ± 0.27
4	8.55	18.55	0.095± 0.01	5.19 ± 0.13	86.16 ± 0.11	−1.36 ± 0.01	16.15 ± 0.23	5.54 ± 0.21	8.79 ± 0.37	7.60 ± 0.88
5	0.00	15.00	0.087 ± 0.00	4.60 ± 0.66	88.31 ± 0.34	−0.57 ± 0.01	11.10 ± 0.49	0.00 ± 0.01	0.00 ± 0.08	0.00 ± 0.12
6	10.00	15.00	0.084 ± 0.01	4.66 ± 0.02	82.62 ± 1.47	−1.12 ± 0.30	20.92 ± 1.47	11.36 ± 1.41	10.94 ± 0.57	8.65 ± 0.07
7	5.00	10.00	0.064 ± 0.01	3.51 ± 0.17	87.13 ± 0.65	−1.00 ± 0.03	14.01 ± 1.18	3.17 ± 0.45	4.94 ± 0.16	4.59 ± 0.14
8	5.00	20.00	0.082 ± 0.00	5.27 ± 0.03	85.75 ± 0.44	−0.99 ± 0.03	16.02 ± 0.40	5.56 ± 0.45	5.89 ± 0.49	3.05 ± 0.12
9	5.00	15.00	0.090 ± 0.01	3.99 ± 0.01	86.03 ± 1.03	−0.97 ± 0.08	15.60 ± 1.45	5.06 ± 1.42	6.49 ± 0.47	4.40 ± 0.21
10	5.00	15.00	0.096 ± 0.01	4.05 ± 0.26	87.16 ± 0.75	−1.08 ± 0.01	14.09 ± 0.88	3.24 ± 0.86	5.49 ± 0.47	4.54 ± 0.21
11	5.00	15.00	0.087 ± 0.00	4.19 ± 0.15	87.03 ± 1.27	−1.03 ± 0.03	14.05 ± 1.57	3.24 ± 0.28	5.99 ± 0.23	4.71 ± 0.16
12	5.00	15.00	0.101 ± 0.01	3.33 ± 0.53	88.13 ± 0.38	−1.00 ± 0.01	12.56 ± 0.83	1.54 ± 0.63	4.65 ± 0.42	4.16 ± 0.32

**Table 6:** Color parameters for commercial IP found by other researchers.

Reference	L		a*		b*	
	Min	Max	Min	Max	Min	Max
Coelho (2005) [40]	17.35	23.86	−4.67	−3.91	14.17	19.41
Mendes (2005) [41]	53.65	59.18	−28.85	−22.40	46.99	56.59
Battiston et al. (2016) [42]	-	38.3	-	−1.76	-	10.81

Min: minimum; Max: maximum.

## Data Availability

The original contributions presented in the study are included in the article, further inquiries can be directed to the corresponding author.
